# Three-dimensional quantitative fracture analysis of tight gas sandstones using industrial computed tomography

**DOI:** 10.1038/s41598-017-01996-7

**Published:** 2017-05-12

**Authors:** Jin Lai, Guiwen Wang, Zhuoying Fan, Jing Chen, Ziqiang Qin, Chengwen Xiao, Shuchen Wang, Xuqiang Fan

**Affiliations:** 1 State Key Laboratory of Petroleum Resources and Prospecting, China University of Petroleum-Beijing, Beijing, 102249 China; 2 College of Geosciences, China University of Petroleum-Beijing, Beijing, 102249 China; 3 Research Institute of Petroleum Exploration and Development, Tarim Oilfield Company, CNPC, Korla, 841000 Xinjiang China; 4 Department of Petroleum Engineering, University of Wyoming, E Lewis Street, Engineering Building, Laramie, Wyoming, 82071-2000 USA

## Abstract

Tight gas sandstone samples are imaged at high resolution industrial X-ray computed tomography (ICT) systems to provide a three-dimensional quantitative characterization of the fracture geometries. Fracture networks are quantitatively analyzed using a combination of 2-D slice analysis and 3-D visualization and counting. The core samples are firstly scanned to produce grayscale slices, and the corresponding fracture area, length, aperture and fracture porosity as well as fracture density were measured. Then the 2-D slices were stacked to create a complete 3-D image using volume-rendering software. The open fractures (vug) are colored cyan whereas the calcite-filled fractures (high density objects) are colored magenta. The surface area and volume of both open fractures and high density fractures are calculated by 3-D counting. Then the fracture porosity and fracture aperture are estimated by 3-D counting. The fracture porosity and aperture from ICT analysis performed at atmospheric pressure are higher than those calculated from image logs at reservoir conditions. At last, the fracture connectivity is determined through comparison of fracture parameters with permeability. Distribution of fracture density and fracture aperture determines the permeability and producibility of tight gas sandstones. ICT has the advantage of performing three dimensional fracture imaging in a non-destructive way.

## Introduction

The Lower Cretaceous Bashijiqike sandstones are the principal gas productive reservoirs in the Kelasu thrust belt, Kuqa depression of Tarim basin^1–3^. However, the Bashijiqike Formation, as a typical tight gas sandstone reservoir, is generally characterized by deep burial depth, low porosity, low permeability and strong heterogeneities^4,5^. Due to the tight nature of the matrix, the well-developed natural fracture systems play a significant role in natural gas transport from the reservoir to the wellbore^2^. Fractures are important in tight formations because they can constitute major paths for fluid flow and increase drainage surface area in oil and gas systems^6^. The successful exploration and efficient development of natural gas in Bashijiqike sandstones depend on the detailed characterization of pore network and fracture systems^2,5,7^. Therefore the characterization of fracture systems is vitally important in exploration, production and development of oil and gas fields^8,9^.

Fractures may appear in all sizes with its aperture being rough and variable^10^. Quantification of fracture geometry remains essential for characterizing transport processes in fractured reservoirs^10,11^. Efforts to quantify fracture geometries and their relationship to permeability have been ongoing for decades^12^. High-resolution industrial X-ray computed tomography (ICT), based on the differential attenuation recording of an X-ray beam through a sample^11^, is a nondestructive laboratory tool that provides *in situ* studies of the three-dimensional distribution of the interior structure of rocks^6,13,14^. ICT is widely used by oil and gas service companies for evaluating the fracture geometries of reservoir rocks to hand-sample size^15,16^. ICT produces two-dimensional slices revealing the interior structures of the reservoir rocks, and the digital data allow three-dimensional measurement of fracture attributes by acquiring a contiguous set of slices^15,17,18^.

This study aims not only at the quantification of the fracture systems, but also at providing a quantitative characterization of fracture attributes by 2-D slice analysis and 3-D volume-rendering. Hand measurements were firstly performed on the 30 core segments, and open fractures as well as calcite-filled fractures are recognized. A series of slices for each segment were produced by CT scanning, and these slices were analyzed using standard image analysis techniques to quantify the fracture area. Vug (open fracture) and high density (calcite-filled) were modeled on each scan slice, and the volume, length, and width of each object (vug or high density) is measured. Sequential contiguous images are then compiled to create 3-D representations by volume rendering. The open fractures are colored cyan whereas the high density objects are colored magenta. Then the surface area and volume of both open fractures and high density fractures are summarized by 3-D modeling. The fracture parameters such as fracture porosity, aperture and density are calculated by a combination of 2-D slice analysis and 3-D visualization and counting, and these fracture parameters are correlated with those derived from the image logs. In order to investigate fractures in the context of deep subsurface fluid flow and hydrocarbon production, quantification of fracture connectivity is then measured by comparing the fracture parameters with core-measured horizontal permeability. Quantitative characterizations of fracture attributes help improve our understanding of fracture connectivity, hydrodynamics, reactivity, and mechanics^16^. The fracture geometries of core samples using ICT in laboratory could be a beneficial complement to the traditional MS/AE (microseismic/acoustic emission) monitoring systems, which could provide real-time fracture information of rockmass^19–22^.

## Results

### ICT analysis

Though with much higher resolution, the Industrial X-ray CT (ICT) is based on the same principles as medical CAT (computed axial tomography) scanning^23^. During scanning, the source sends out an X-ray beam with intensity *I*_0_^24^. As the X-ray beam pass through the object being scanned, the signal is attenuated by scattering and absorption, therefore an X-ray beam with a lower intensity *I* is measured by the detector^23^. The attenuation of the X-ray beam through a homogeneous object can be described by Beer’s Law^13,15,17,18,23,25,26^:

1 $$I={I}_{0}{{\rm{e}}}^{(-\mu x)}$$

where *I*_0_ is the initial intensity of the X-ray beam, *I* is the final intensities of the X-ray beam after it has passed through a length *x* of material, and *μ* is the linear attenuation coefficient^18,23^.

**Figure 1:**
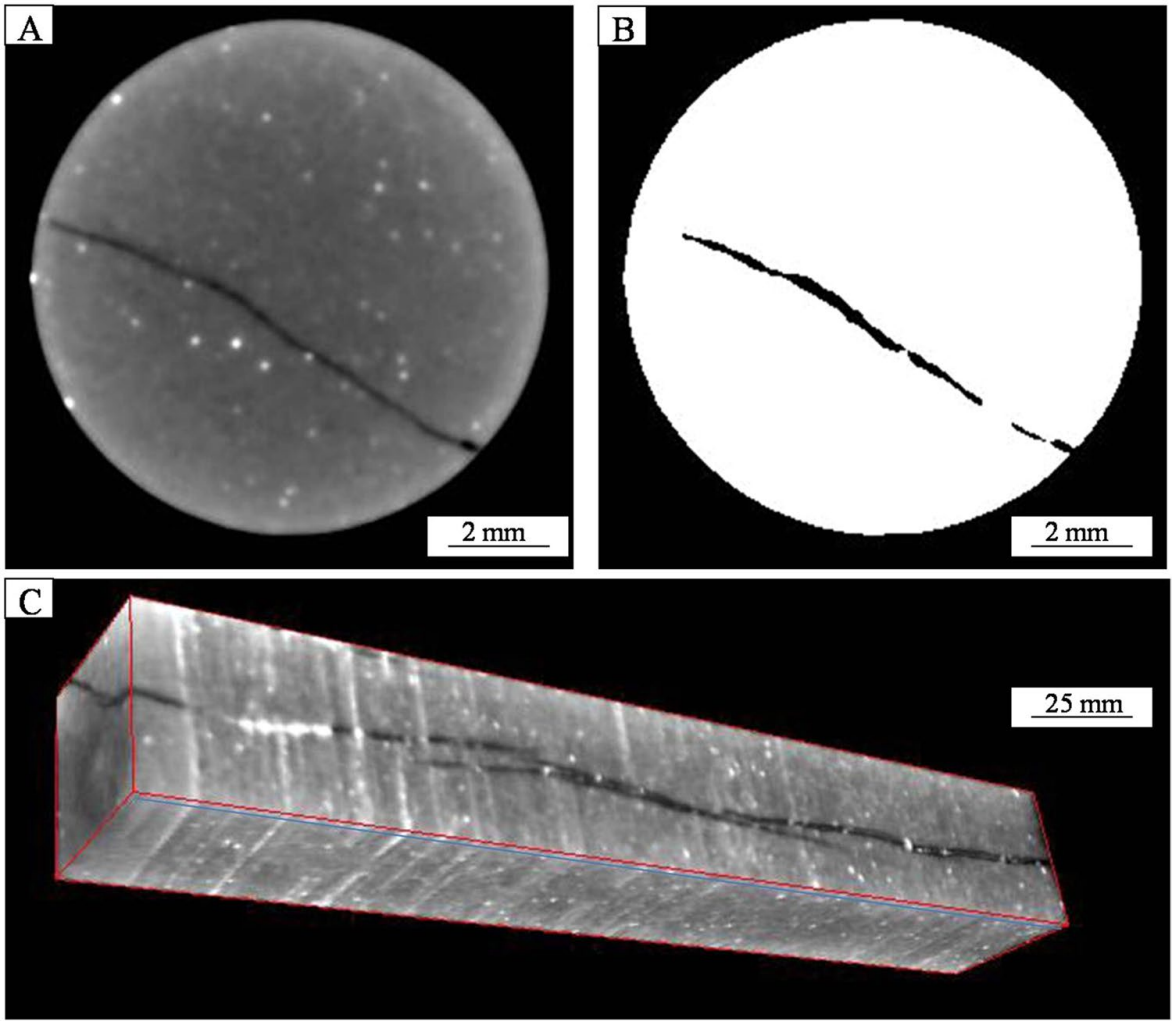
Example of ICT 2D slices (**A,B**) and the reconstructed 3D volume (**C**) by a stack of contiguous slices.

An industrial X-ray CT scanner, which allows using of higher energy X-rays and longer dosage times^23^, was used in this study to obtain high-resolution volumetric reconstructions of fractures. The resulting data (X-ray with intensity *I*) are firstly reconstructed to create two-dimensional images (“slice”) of the object (e.g. Fig. 1A,B)^23^. Each slice represents a finite thickness (0.3 mm in this study) of material^23^. The scanner produces images of 1024 × 1024 pixels with a pixel resolution of 140 × 140 μm^2^. By gathering a set of continuous slices, a complete 3-D volume can be reconstructed, allowing three-dimensional inspection and measurement of features of interest (Fig. 1C)^23^. Therefore the volumetric scans consisted of multiple 0.3-mm thick cross-sectional stacked slices. The pixels in CT images are referred to as voxels (volume elements)^23^, and each voxel represents a volumetric element and consists of a CT number that is related to the density and atomic number^25^. The CT number, or the X-ray attenuation number, can be derived from the linear attenuation coefficient of X-ray^25,27^.

2 $$C{T}_{number}=(\frac{{\mu }_{c}-{\mu }_{w}}{{\mu }_{w}})\times 1000$$

where *μ*_c_ is the calculated X-ray attenuation coefficient and *μ*_w_ is the attenuation coefficient for water, and 1000 is the proportionality coefficient^25,27^. Similar to medical CT systems, industrial CT systems also use the Hounsfield Unit (HU), in which air has a value of 0, water of 1000, and aluminum of 2700^17^.

When a given X-ray energy is used, the grayscales in the CT images reflect the relative linear X-ray attenuation coefficient^13,23^. By convention, increasing brightness in CT image corresponds to increasing effective attenuation, which in turn reflects increasing density or atomic number^13^. Compared with medical scanners, high-resolution ICT scanning is capable of imaging individual pore and fracture at much higher resolutions, enabling more detailed analysis of fractures at the scale of a petrographic thin section at low to intermediate magnification^15,17^.

If the modeling on the CT images were performed without cropping the cylindrical core samples, the fracture will run out of the core; therefore, every slice in the data set is cropped. In addition, core surface effects created by the external boundary must be eliminated to determine fracture porosity. In fact, the region captured in a CT scan is generally larger than the diameter of the core segments, therefore the raw CT image is cropped into the “region of interest” (ROI). The ROI is a circle area wiping off the outer edges of the sample in the CT image (Fig. 1)^14^. The circle area in Fig. 1A,B is the area of original sampling whereas the 3-D modeling volume is assembled by cropped ones (Fig. 1C). The use of ROI can avoid the interferences of beam hardening to some extent^14^. That’s why the CT slices in Fig. 1 are circular in shape, while the reconstructed 3-D modeling volume is cubic in shape. Each voxel represents a volumetric element of 0.14 × 0.14 × 0.3 mm. In fact, the Volume of Interest is a rectangular polyhedron that is cut out of the middle of the core in this study and this allows us to remove the surface effects, and it makes the thresholding (binary images) easier. The relationship between resolution and lower limit of fracture length and aperture is dependent on the sample size and the differences between the matrix and fracture, and 0.1 mm is about the cut-off that can be typically observed.

Hand measurements of each fracture length and the average aperture were performed on each segment of core (the resulted CT image). The fracture azimuths and dips were also measured on the circumferential CAT (computed axial tomography) scan images. All core segments were scanned at room temperature and atmospheric pressure by an X-ray CT to evaluate fracture network. Each sample was scanned 458–936 times along its axial direction to obtain consecutive gray-scale images^28^. The CT slice thicknesses (0.3 mm) were retained to enhance both resolution and contrast. CT data acquisition procedures such as sample preparation, calibration, collection and reconstruction are following the methodology of Ketcham and Carlson (2001) and Kyle and Ketcham (2015)^17,18^.

As discussed above, macropores and fracture systems can be distinguished based on the discrepancy of CT numbers^28^. In a typical binary black–white image, each voxel corresponds to a gray scale that is correlated with a CT number^28^. Void space (fractures or macropores) with low CT number corresponds to the black color; high density minerals (calcite or hematite) with high CT number correspond to the white; and mineral matters with medium CT number correspond to the gray color (Fig. 1C)^28^.

### Image logs and core observations

**Figure 2:**
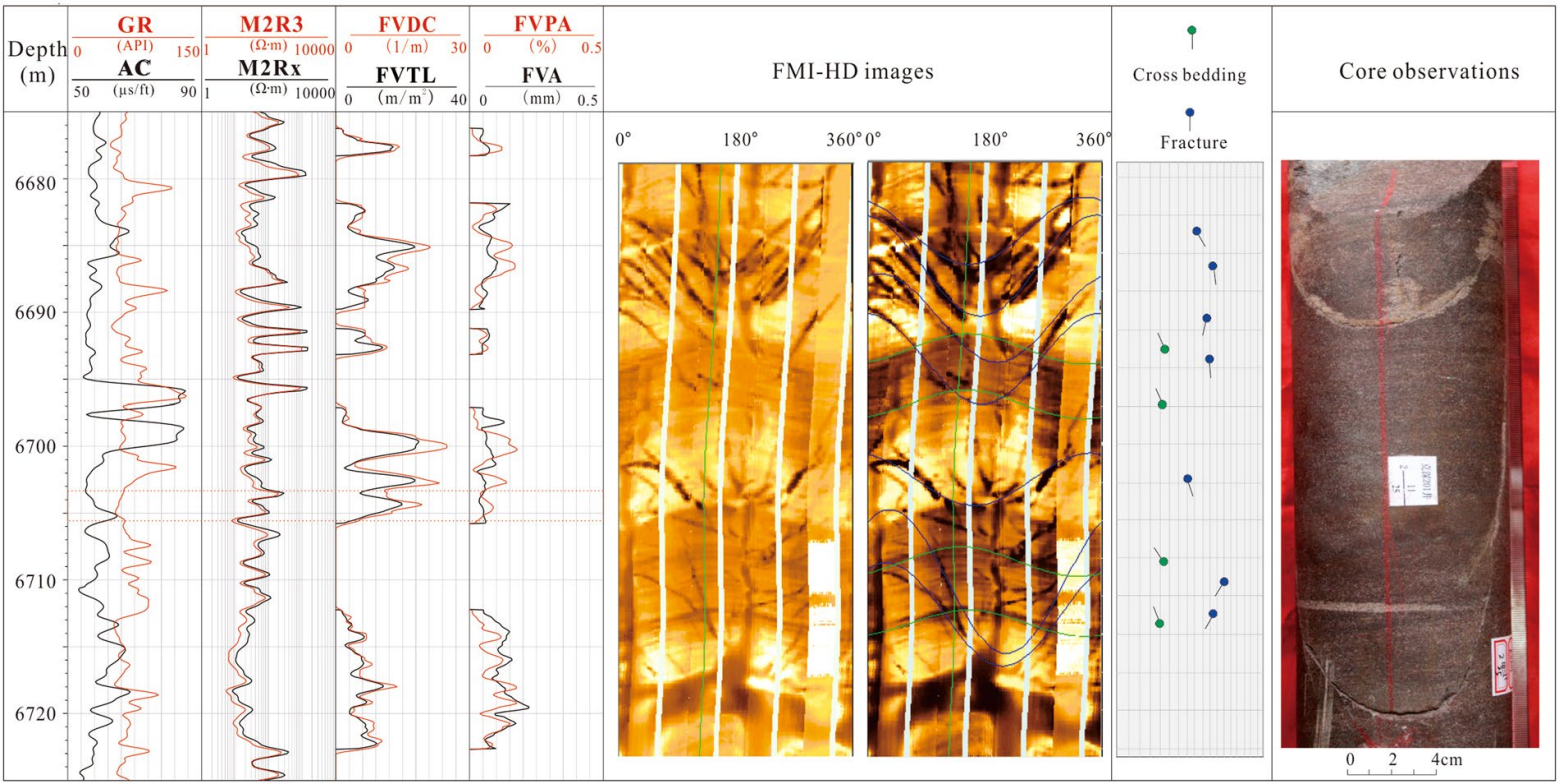
Fracture analysis using a combination of cores and image logs (GR: Gamma Ray; M2R3 and M2Rx: high definition induction log; AC: Acoustic log; FVDC: fracture density; FVTL: fracture length; FVPA: fracture porosity; FVA: fracture aperture).

Open fractures are evident on borehole FMI image logs in wells drilled with water-based muds^8^. In case the mud is conductive, open fractures can be picked out by the dark continuous sinusoidal wave appearance in the image log (Fig. 2)^29–31^. By detailed core observations and image log analysis, natural fracture systems, which play an important role in fluid flow and hydrocarbon production, are well developed in the Bashijiqike tight sandstones (Fig. 2)^4,30^. A total of 7 open fractures (purple line) could be traced from the image logs in Fig. 2, and the dip angles of these fractures are high (>45°). The core observations support the interpretation of image logs (Fig. 2).

Fracture attributes such as fracture dip, orientation, fracture density (FVDC), fracture aperture (FVA), fracture porosity (FVPA) can be determined by interpreting the image logs^9,31–33^. These fracture parameters such as porosity and aperture can also be compared and calibrated with core observations^32^.

Fracture porosity from electrical borehole scans is deduced as following^34^:

3 $$FVPA=\frac{1}{2\pi RLC}\sum _{i=1}^{n}{L}_{i}{W}_{i}$$

where FVPA is the fracture porosity (%), *R* is the borehole radius (m), *L* is the length of statistical interval (m), C is the coverage ratio of image logs, *L*_i_ is the length of *i*^th^ fracture (m), and *W*_i_ is the fracture aperture (mm).

The fracture aperture, which is the width of the open part of a fracture measured at a right angle to the fracture walls^8^, can be calculated by the image logs according to the formula by Luthi and Souhaité (1990)^34^:

4 $$FVA=aA{R}_{m}^{b}{R}_{xo}^{1-b}$$

where the fracture aperture is expressed as a function of the integrated additional current A (m^2^), the resistivity of invaded zone, *R*_xo_ (Ω·m), and the resistivity of the drilling mud *R*_m_ (Ω·m), through the tool-dependent coefficients *a* and *b*^34,35^.

Fracture density (FVDC) is defined as the numbers of fractures per unit length or cumulative fracture trace lengths per unit area or cumulative fracture areas per unit volume^8^, and it can be derived from the image logs as follows (Eq. (5)):

5 $$FVDC=\frac{1}{L}\sum _{i=1}^{n}{L}_{i}$$

After manually picking out all the open fractures in the image logs, the mode “Export Fracture Channels” in the software Geoframe was used^31^, and then all these fracture parameters can be derived from the image processing (Fig. 2). There is no doubt that the higher the fracture density, and the larger the fracture aperture, the higher the fracture porosity will be. The well KS 201 is taken as an example here, and the fracture porosity of Bashjiqike tight gas sandstones in this well is in the range from 0 to 0.56% with an average of 0.04%. By contrast, the fracture aperture ranges from 0 to 0.6 mm with an average of 0.05 mm. The fracture density varies from 0 to 25.7 m^−1^, and averages as 3.2 m^−1^ (Fig. 2)^31^.

### Hand measurements

**Figure 3:**
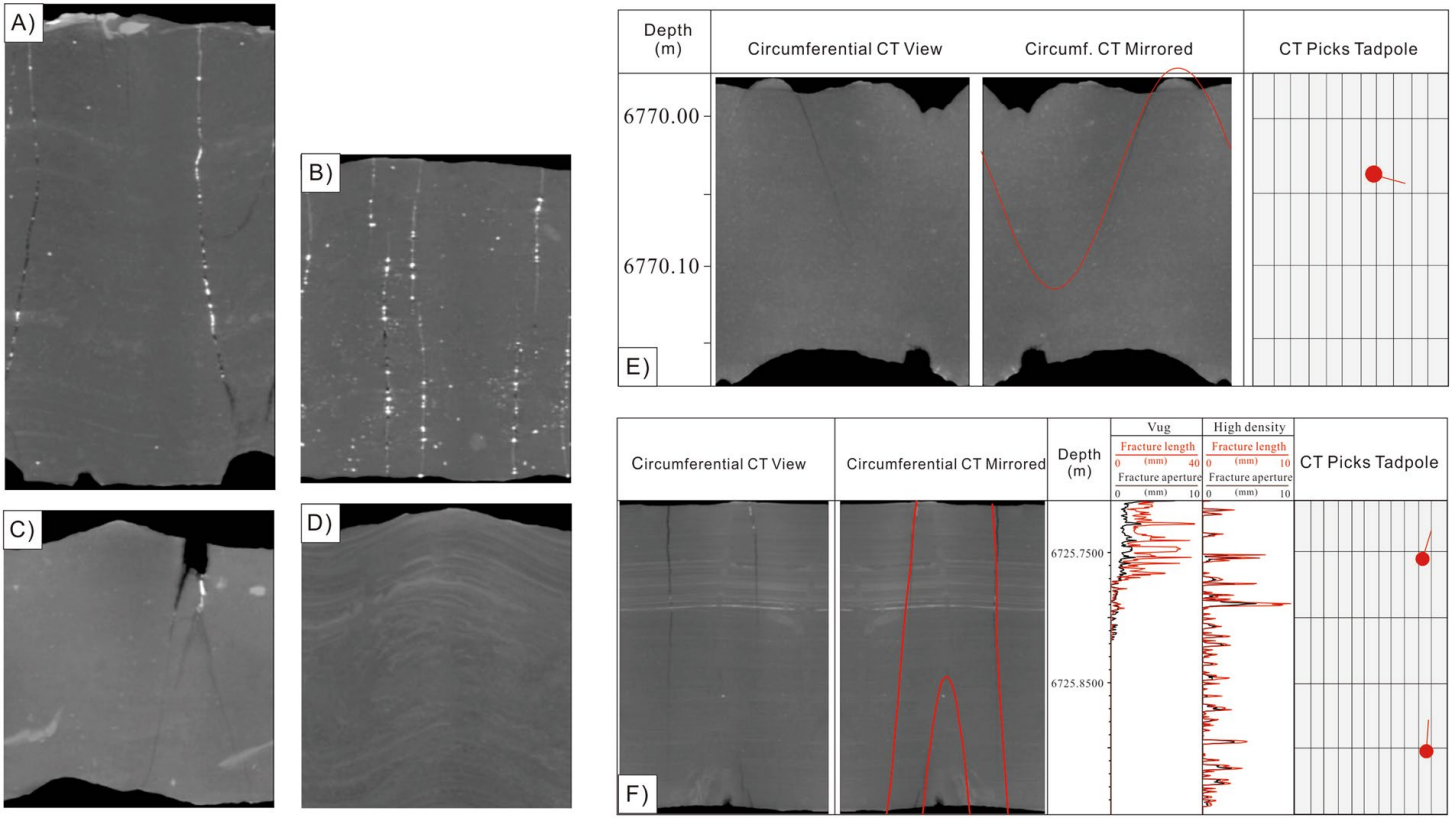
Hand measurements and ICT analysis of core segments. (**A**) A long, calcite-filled, hair-line (0.1 mm), extensional fracture with a lower termination is observed within the core. Well Keshen 2-2-8; 6718.42-6718.71 m; (**B**) Segment 6680.42: four, thin-aperture (0.4–0.8 mm), calcite-filled, extensional fractures that are oblique to bedding. (**C**) Segment 6767.5 m: Two coring induced petal fractures on the edge of the core. (**D**) Segment 6734.83; No fractures. (**E**) The directional fracture measurements (azimuth and dip) were conducted on the circumferential CAT scan images. (**F**) Two-dimensional slice analysis of fracture length and aperture for both vugs (open fracture) and high density (closed fracture) objects.

A visual inspection of each fracture density and the average aperture were performed on each core segment. The fracture types observed in these core segments consist of extensional fractures (Fig. 3A,B) and the coring induced petal fractures (Fig. 3C). In addition, there are no fractures could be detected in some samples (Fig. 3D). Fractures are sometimes filled with calcites (Fig. 3A,B). The directional fracture measurements (azimuth and dip) were conducted on the circumferential CAT scan images. The projection or the sine curve fracture picks are conducted on a mirrored circumferential CAT scan view; so that, it appears that you are looking from within the well bore versus looking at the surface of the core (Fig. 3E). These mirrored images should look like and be correlatable to downhole image logs like FMI log (Fig. 3E).

### Two-Dimensional slice measurements

Individual CT images are generally referred to as two-dimensional (2-D) slices^15^, and these CT images are reconstructed based on the CT_number_ which depends on the density and the atomic number^24^. Each CT scanning produces a series of slices, and these CT slices were analyzed using standard image analysis techniques to quantify the fracture area within each slice^27^. In order to achieve a high accuracy, segmentation was conducted on each CT image to make sure that all void spaces representing open fractures are clearly distinguished from mineral phases^14,16^. For example, the fracture appeared in the slice in Fig. 1B can be segmented into four parts labeled as 1, 2, 3 and 4 (Fig. 1B). Therefore the number of 2-D fracture measurements is larger than the number of slices. For example, the 588 slices of Sample 10 had been measured for 1052 times to separate fractures based on more robust criteria rather than simply the CT values.

**Figure 4:**
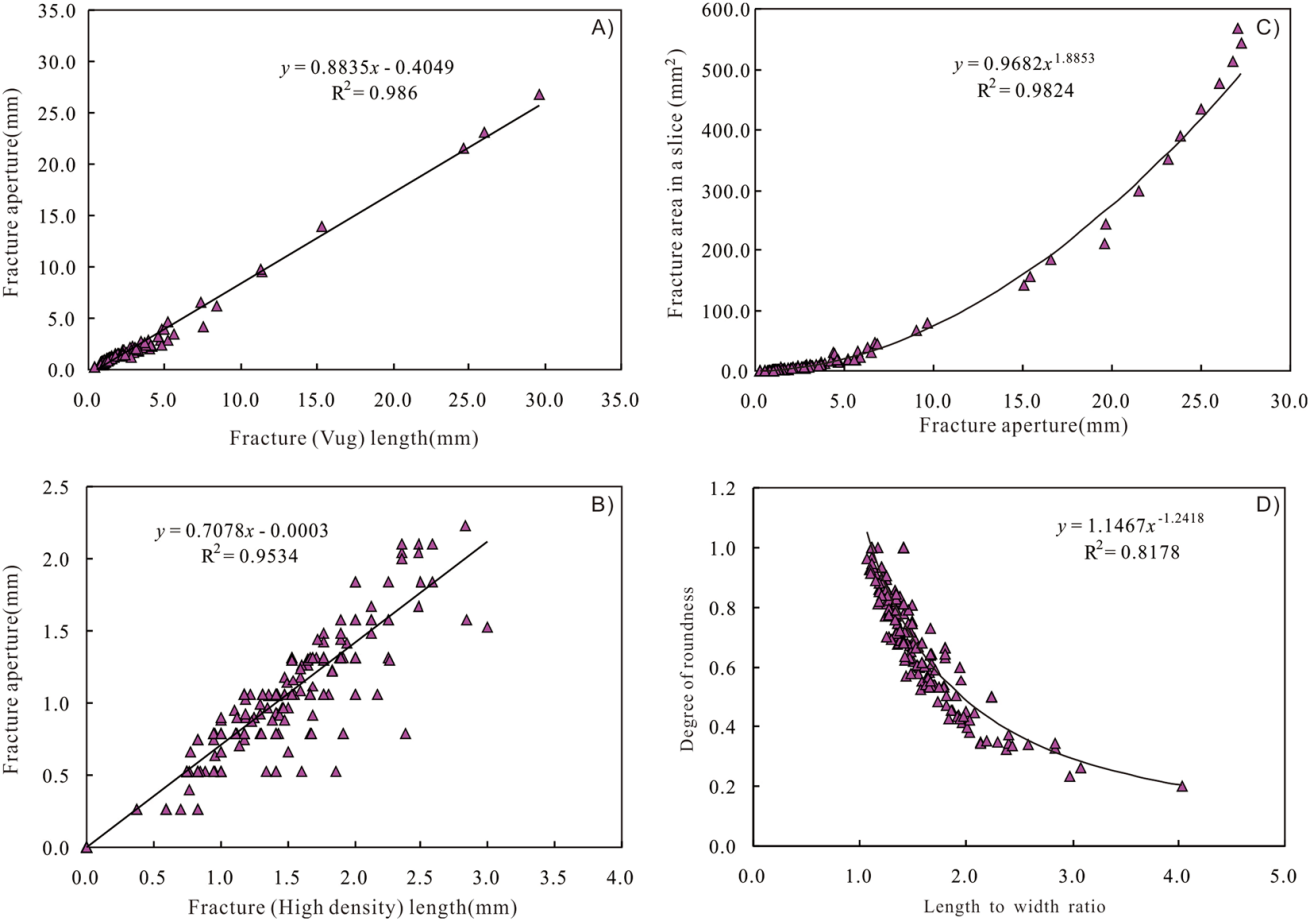
Crossplots showing the relationships between (**A**) open fracture aperture and fracture length; (**B**) calcite-filled fracture aperture and fracture length; (**C**) fracture area in a slice and fracture aperture; (**D**) Crossplot of degree of roundness and length to width ratio for open fractures.

For each CT model on every slice, the area, length, and width of each object (vug or high density) is measured (Fig. 3F). The average fracture length and width (aperture) of each slice is also summarized (Fig. 3F; Supplementary Table 1). For example, a total of 803 slices were analyzed for the Sample 7, among these slices, there are 110 slices encountered the open fractures, and a total of 275 small fractures can be picked out by segmentation techniques. In contrast, the calcite-filled fractures are detected in the 171 slices (Supplementary Table 1). The open fracture length derived from 2-D analysis is in the range from 0.37 mm to 29.64 mm with an average of 3.20 mm, whereas the fracture aperture by 2-D analysis ranges from 0.26 mm to 26.8 mm, and averages as 2.43 mm. The regression analysis results show that the open fracture aperture is positively correlated with fracture length with a high correlation coefficient of 0.98 (Fig. 4A). Likewise, the length of the calcite filled fractures is also strongly positively correlated with the fracture width or aperture (Fig. 4B). In fact, for all of the core segments, the length of both open fractures and calcite-filled (high density) fractures is positively correlated with the fracture aperture. Fracture aperture distribution is essential for description of fracture network geometry and characterizing transport processes in fractured rock matrices^11^. The fracture aperture determines the total area of fractures in the 2-D slices (Fig. 4C). The 275 fractures in Sample 7 are generally small and range in length from 0.37 mm to 31.05 mm, in aperture from 0.26 to 27.27 mm, and have an average length/aperture ratio of about 1.52. The ratio between open fracture length and aperture (length to width ratio), which ranges from 1.07 to 4.04 with an average of 1.52, shows good positive relationships with the degree of roundness (Fig. 4D).

### Three-Dimensional modeling

The main advantage of X-ray CT scan in rock characterization is that the data are obtained in three-dimensions^32^. The 3-D fracture modeling is indispensable for interpreting transport processes in rocks with regard to the 3-D connectivity of the fracture network^11^. A series of 2-D slices can be stacked to create a complete 3-D volume matrix data^14^. As discussed above, the fundamental CT data unit is the volume element (voxel), which corresponds to the volume bounded by the edges of a pixel and the thickness of the slice^15^.

**Figure 5:**
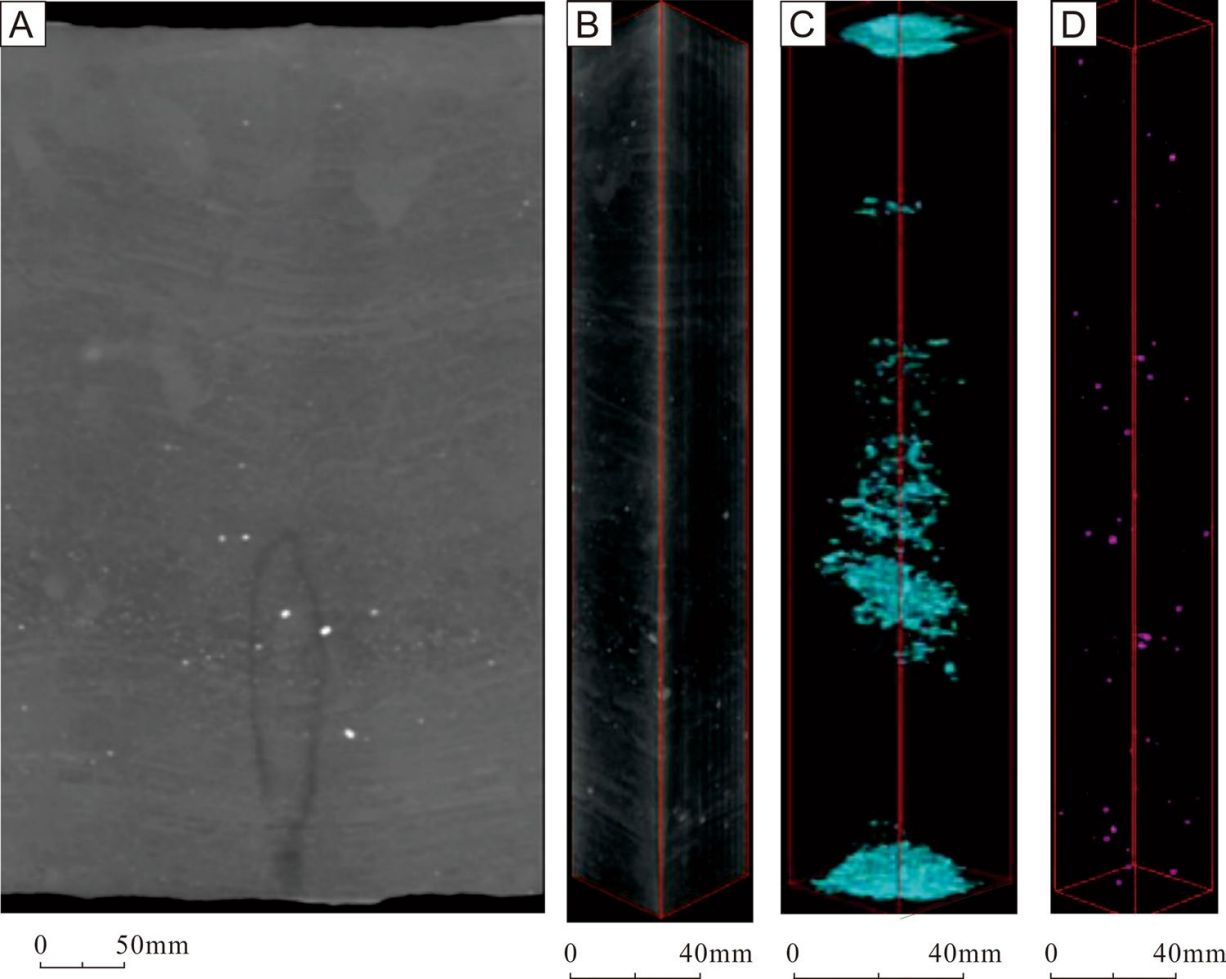
Interior structure reconstructed by ICT analysis. (**A**) ICT Circumferential View; (**B**) Images reconstructed by computed tomography; (**C**) 3-D Volume-rendering of open fractures (colored cyan); (**D**) 3-D Volume-rendering of calcite-filled fractures.

**Figure 6:**
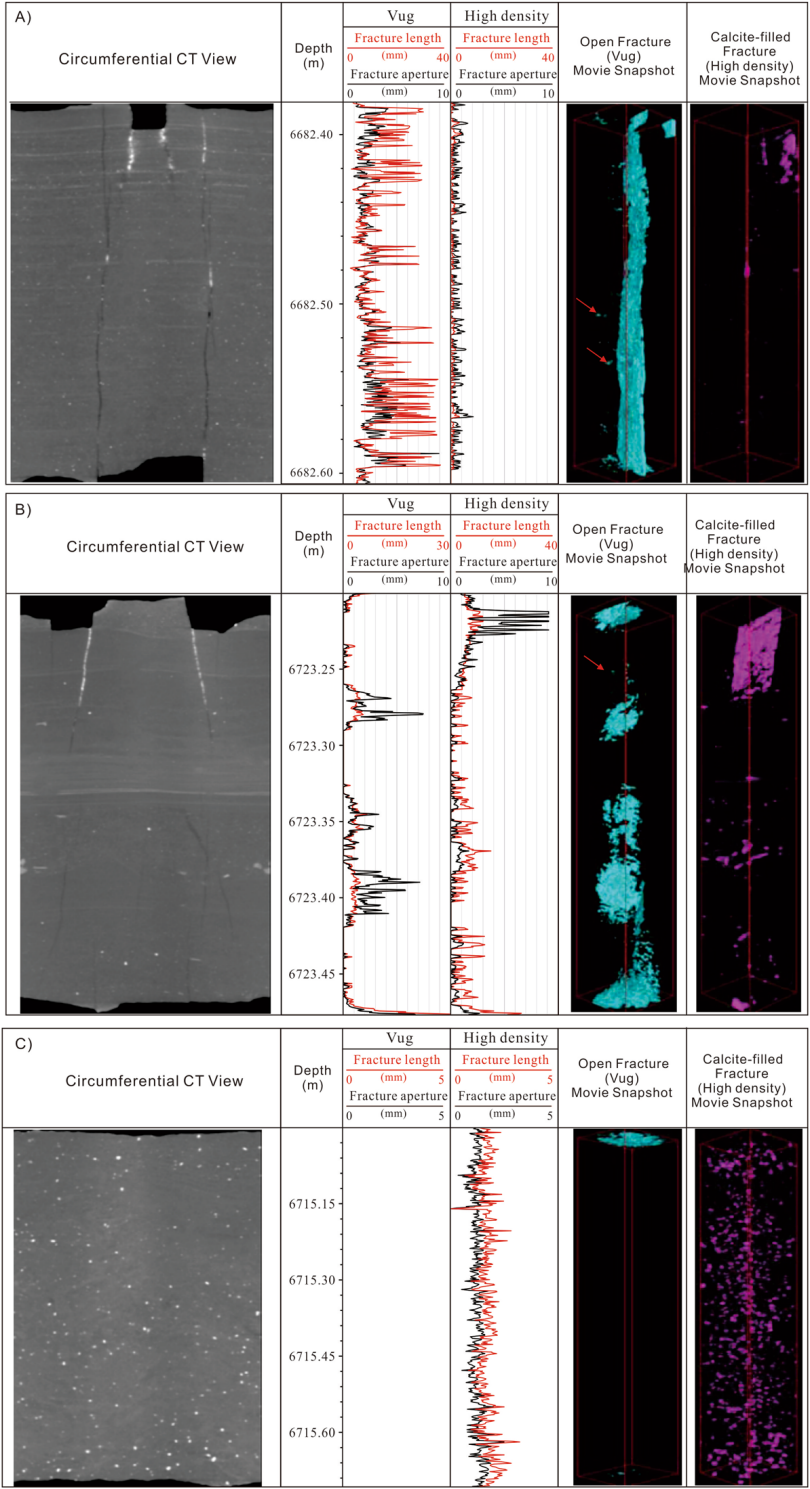
Three-dimensional volume rendering of fractures for Sample (**A**) segment 6682.38; (**B**) segment 6723.2 m; (**C**) segment 6715.00 m. Note the locally distributed pores (red arrow).

Each model is 3-D rendered into a movie using volume-rendering software, and the models are generated by making threshold density cutoffs for each model. Volume rendering, in which each grayscale value in the data set is assigned a color and an opacity value according to its CT number, is the most powerful three-dimensional visualization technique (Fig. 5)^23^. There is no single appropriate CT value that marks the boundaries between rock materials and voids^24^, however, volume rendering helps to circumvent this difficulty by showing different fractures in different colors and intensities^17^. ICT could be used to distinguish mineral-filled fractures from open fractures since CT values increase with density and atomic number^27^. A very low and a high density model were performed on each slice, and each object on a slice was measured. The models were engineered to represent the open fracture (Vug) and the calcite-filled fracture (high density) network (Figs 5 and 6A). In this study, the fractures have been made more visible by false coloring, and all of the open fractures are colored cyan whereas all of the high density objects are colored magenta for each segment (Figs 5 and 6A).

Another example image of a fractured core fragment is shown in Fig. 6B, and the 3-D volume-rendering reconstruction of the stacked slices to reveal the fractures and macropores is shown in Fig. 6B. The low density model is the interface between air and the fracture. The high density objects are calcite-filled fractures, and the very high density hematite nodules (Fig. 6A). Another typical 3-D reconstruction result for natural fracture systems of Sample 24 are shown in Fig. 6B. Compared with the X-ray diffraction data^36^, it could be concluded that the white circular particles in the image are mainly calcite nodules, with possibly minor hematite, and the more dispersed, slightly darker colored surrounding mineral components are mainly framework grains such as quartz and feldspar (Fig. 6A). The fracture partly filled with calcite is clearly visible in the CAT scan slice (Fig. 6B). There are obvious differences between open and calcite-filled fractures. In Fig. 6A, two vertical open fractures can be observed, and the fractures can be detected by all the 2-D slices. The well-developed natural fracture network appears as cyan bands on the movie snapshot (Fig. 6A). In contrast, the areas partly occluded by the calcites can be recognized as magenta colors (Fig. 6A). Figure 6B shows the 3-D volume-rendering reconstruction of the 922 stacked slices, representing a complete view (a movie snapshot) of the fracture. Among them, the open fractures appear in about 478 slices of them. In contrast, the high density objects (calcite-filled fractures) have been detected in 136 slices (Supplementary Table 1; Fig. 6B). In the core segments without fractures, only magenta colored calcite or hematite filled nodules can be observed in the movie snapshot (Fig. 6C).

**Figure 7:**
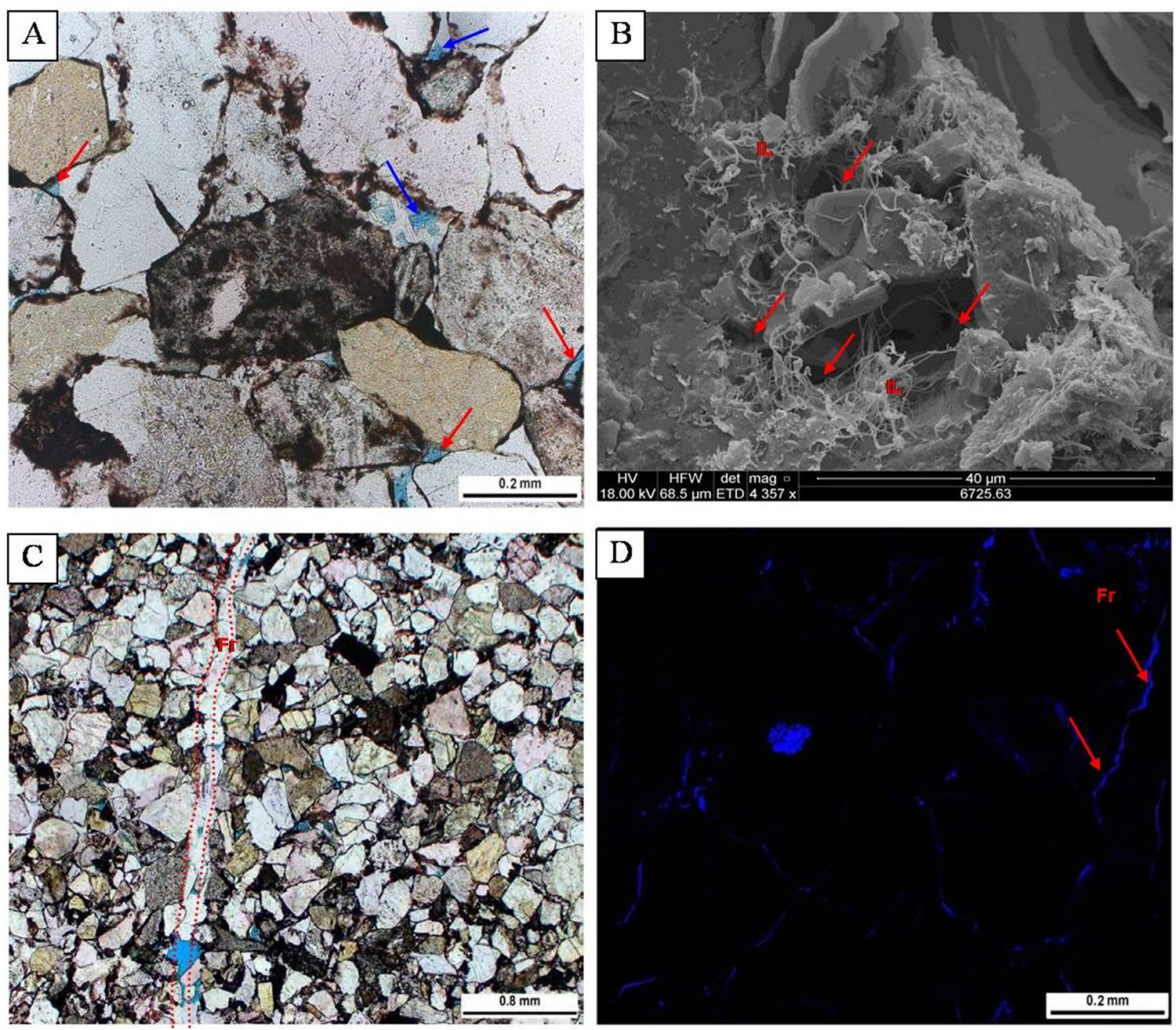
Photomicrographs showing the pore systems of the sandstones. (**A**) Intergranular pores (red arrow) and intragranular pores (blue arrow). (**B**) Miicropores (red arrow) associated with the illite (IL). (**C**) Natural fractures (red dashed lines). (**D**) Fractures (Fr) are present.

It should be noted that the 3-D reconstruction suggests that there are no obvious pores (<100 μm) within the core fragments (Fig. 6A,B), however this phenomenon is attribute to the finite resolution of the imaging to some extent. The core segments used in this study are hand-scale size, and, due to the large sample size, the image resolution by CT is lower. Previous studies confirm that the pore system of Bashijiqike sandstones are dominantly intragranular pores and micropores associated with feldspar dissolution and authigenic clay minerals, whereas the intergranular macropores are rarely observed (Fig. 7A,B)^5,33,36–38^. The macropores, which shows cyan spots in the 3-D models, have a heterogeneous distribution with poor connectivity (Fig. 6A,B). Therefore only the fractures and minor amounts of large open macropores can be modeled in this study. In fact, there are also microfractures (Fig. 7C) below the resolution of the CT modeling^3^. X-ray CT images in this study are unable to map some microfractures that can be observed with SEM and thin sections (Fig. 7C,D)^6^.

## Discussions

From Fig. 6A,B it is clear that most fractures, which can be observed in the hand measurements, can be detected by CT modeling^39^. The 3-D volume-rendering method ensures that fractures in all orientations could be detected, which is a clear advantage over the 2-D methods, especially for fractures that are near-parallel to the slicing direction^40^. ICT is able to nondestructively characterize the 3-D distribution of macrofractures^11^. Resolution from X-ray CT can detect fracture attributes that well-logging geophysical methods cannot^6^, since the CT could provide nondestructive three-dimensional visualization and characterization^17^. However, CT is not yet adapted for detecting microcrack networks, especially for handscale samples^11^. Increased resolution will often imply a reduction of the size of the scanned rocks^6^. Due to the large sample size and lower resolution in this study, it is not easy to distinguish the micropores and microfractures in this 3-D data, only macropores and macrofractures of the samples can be identified from the CT scanning.

Beyond visualization comes the need to extract quantitative parameters about fractures in the scanned volumes^15^. ICT instruments provide resolution on the order of 100–200 μm for the hand-scale core segments used in this study. As discussed above, the 3-D reconstruction of 2-D grayscale slices provides an intuitive presentation of the spatial dispositions of pores, and fractures^14^. In this section, the total fracture volume (%) in each slice and the average length of the fractures on each slice are derived from the ICT scanning. In addition, comprehensive analysis is performed using a combination of 2-D slice analysis and 3-D volume-rendering to quantitatively evaluate fracture parameters such as fracture porosity, fracture aperture, and fracture density.

Fracture porosity can be derived from both the 2-D slice measurements and 3-D volume-rendering. Fracture porosity is by definition the fraction of fracture volume versus total rock volume. The total volume of each rock segment is known (Supplementary Table 1). By 3-D volume rendering the total volume of open fractures could also be obtained, and therefore the fracture porosity can be calculated. The 3-D fracture porosity (*P*_3D_) reveals a range from 0 to 4.34%, and has an average of 0.46% (Supplementary Table 1). Likewise, the porosity of calcite-filled fractures could also be calculated. The porosity of these closed fractures shows a wide range from 0 to 14.79% with an average of 2.02% (Supplementary Table 1). A conclusion could be drawn that there are a large number of fractures are filled with the high density calcites.

In the 2-D slice analysis, open fracture porosity (*P*_2D_) equals to ratio of the total fracture area (mm^2^) versus total area analyzed (mm^2^). The analysis results show that the 2-D (open) fracture porosity is in the range from 0 to 4.33% with an average of 0.46% (Supplementary Table 1). This result is in accordance with the fracture porosity determined from 3-D modeling.

Aperture is a key parameter for the estimation of fracture porosity and permeability^41^. As previously discussed, CT numbers for each voxel depends primarily on the density of the material, and the CT value increases with density^42^. Therefore the presence of a fracture can be detected in the CT images for an apparent density reduction, even if the fracture aperture is smaller than the voxel^42^. By counting the number of voxels that have a CT number lower than the rock materials and multiplying them by the size of each voxel, the total volume of the open fracture can be calculated and therefore the average fracture aperture of the whole rock sample can be estimated^43^. For example, the total open fracture volume for Sample 10 was counted to be 608.767 mm^3^ (29337 volume voxels) by 3-D modeling, which for the scanned area of 3165.592 mm^2^ (18615 surface pixels) gives an average fracture aperture of 192 μm^43^. Likewise, the average aperture of high density (calcite-filled) fracture is also calculated (Fig. 6A,B). The calculated fracture (both open and high density) apertures of every core samples are presented in Supplementary Table 1. The Supplementary Table 1 summarizes the total volumes of the low and high density models and the average lengths and widths for each segment. Therefore the 3-D aperture distributions of fractures are precisely visualized using 3-D volume-rendering^44^. For example, most of the fractures in Fig. 6A contain apertures larger than 2 mm, and large aperture regions up to 4 mm are visible on bottom edge of the fracture (Fig. 6A). In contrast, the fracture apertures in Fig. 6B are generally less than 2 mm, and the middle parts (6728.23–6728.26 m and 6728.28–6728.33 m) of the fractures appear closed (Fig. 6B).

By comparison with the fracture parameters derived from the FMI-HD image logs, both the fracture porosities and fracture apertures from CT scan calculation are about an order of magnitude higher than those derived from FMI-HD image logs. On one hand, the ICT analysis especially the 3-D volume-rendering can detect more fractures than the image logs do, therefore the fracture parameter should be higher than those from image logs. On the other hand ICT scanning under atmospheric stress condition in this study facilitates locating open fractures, but does not yield the true surface fracture apertures at depth^6^. The fractures have an obvious sensitivity to stress^45^. Long fractures cause the core to fall apart, and core recovery is very low in intensely fractured intervals and the stress release when the core is taken to the surface will affect the fracture apertures^41^. The aperture and therefore the porosity of fractures will be much smaller at reservoir conditions than that at surface conditions^45^. However, the fracture geometries of laboratory core samples using ICT could be a beneficial complement to the well-logging geophysical methods and MS/AE monitoring systems.

Fracture density is a basic parameter describing how fracture networks evolve as a function of surface age^11^. Fracture density estimation is an indisputable challenge in fractured reservoir characterization. In the CT scanning, since the fracture network was skeletonized, fracture densities can be derived from the 2-D slices. Fracture density (m^–1^) is estimated by dividing the total fracture length in the slice (mm) by the total area of the image (mm^2^) in this study (Eq. (6))^11^.

6 $$D=\frac{N\times L}{S}$$

where *N* is the number of pixels forming the whole skeleton of fractures, *L* is the pixel size (mm), and *S* is the area of the considered image (mm^2^)^11^.

**Figure 8:**
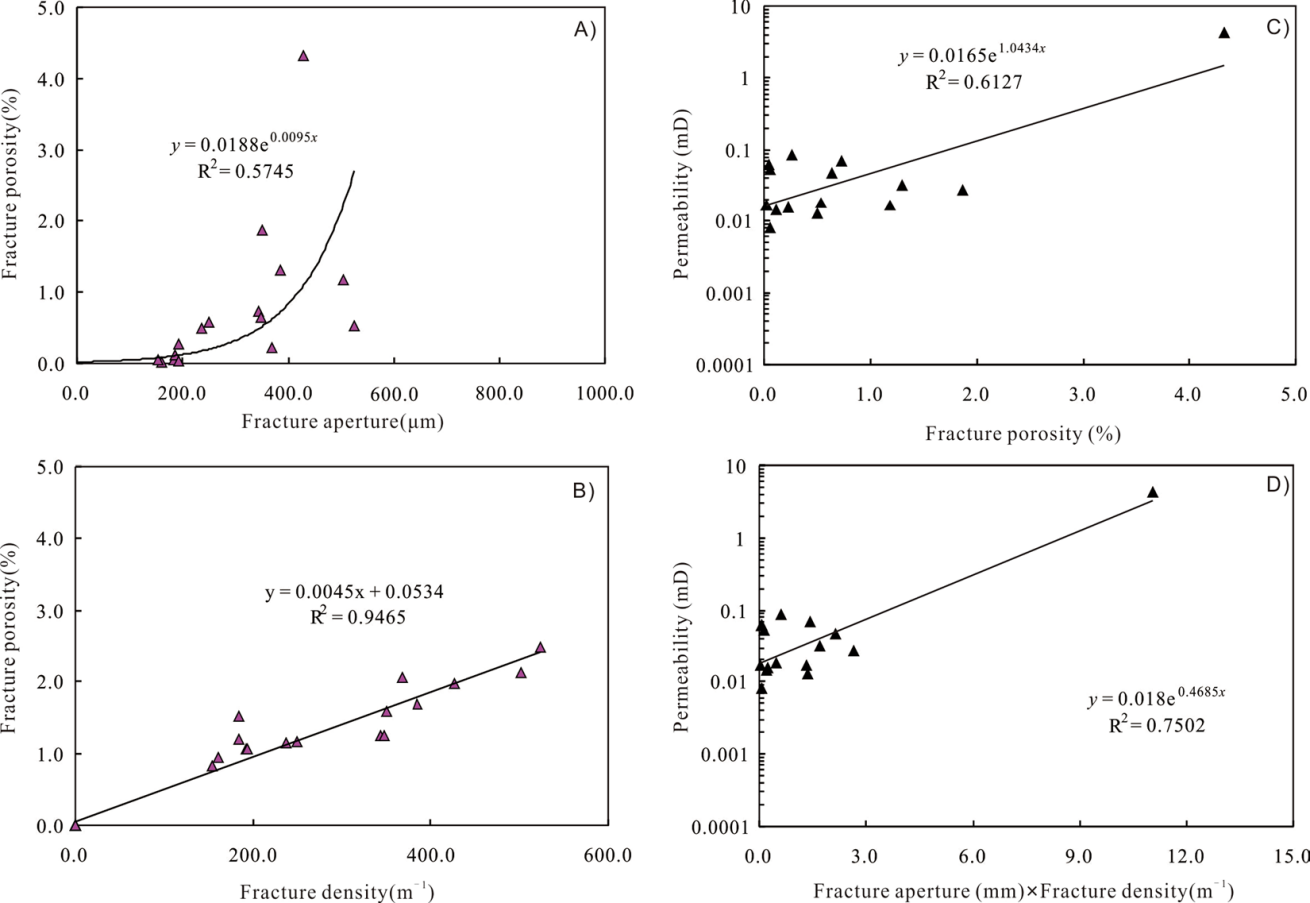
(**A**) Plot of fracture porosity versus facture aperture; (**B**) Plot of fracture porosity fracture density; (**C**) Plot of horizontal permeability versus facture porosity; (**D**) Plot of horizontal permeability versus fracture aperture (mm) multiplied by fracture density (m^−1^).

The calculated fracture density from 2-D slice analysis is in the range from 0.2 to 25.9 m^–1^ with an average of 4.40 m^–1^ (Supplementary Table 1), which is correlatable with the fracture density (FVDC) derived from image logs in Section “Image logs and core observations”. However, an average fracture density of 4.40 m^−1^ is a little higher than fracture density from image logs. Likewise, the 3-D fracture density, which is defined as the area of fractures within a unit volume of rock^41,46^, can be derived from the 3-D volume-rendering. However, it can be concluded from the definition that this 3-D fracture density is the inverse of fracture aperture from 3-D counting. Regression analysis shows that open fracture porosity is generally dependent on both the fracture density and the fracture aperture from 3-D analysis (Fig. 8A,B).

Fractures are the most important fluid pathways for fluid extraction and injection^47^. A thorough understanding of the fracture geometry is of great importance to investigate fractures in the context of hydrocarbon production^16,47^. Effective development of oil/gas fractured reservoirs requires a deep understanding of fracture flow characteristics^42^. Understanding of the 3-D fracture geometry using the 3-D visualization technique, including length, spacing, orientation, and aperture distributions, will provide important insights into the fracture connectivity analysis^48^. Fluid flow in reservoir rocks is often dominated by the highly permeable pathways provided by fractures^49^. Characterizing and understanding the pore structure and the fractures are essential for maintaining and enhancing oil and gas recovery^50^.

Fractures can enhance reservoir permeability by even more than two orders of magnitude, and they play an important role in natural gas accumulation^﻿51,52^. In order for quantification of fracture connectivity from fracture geometry data, the horizontal permeabilities of these full-diameter core segment samples are calibrated with the fracture parameters derived from ICT analysis. Regression analysis shows that no evident relationship is observed between core-measured porosity and fracture porosity, representing that the total porosity is mainly dependent on the matrix porosity rather than fracture porosity. However, the exponential trendline with a regression coefficient R^2^ > 0.61 supports a relative good relationship between horizontal permeability and fracture porosity (Fig. 8C). In addition, moderate exponential relationship is observed between permeability (13.66 MPa) and fracture aperture (mm) multiplied by fracture density (m^−1^) with a regression coefficient of 75% (Fig. 8D). Distribution of fracture density and size of fracture apertures impacts fluid flow. Fractures are the important flow pathways that determine the permeability and producibility of tight gas sandstones.

## Methods

The Schlumberger’s FMI (Formation MicroImager) and FMI-HD (high-definition FMI) borehole image logs were used in this study to help subsurface fracture analysis. The Schlumberger’s software Geoframe was used to build up a “pseudo-picture” of the wellbore according to the electrical resistivity contrasts^53^. Pre-processes such as speed correction, eccentering correction, normalization and depth shift had been done in the Schlumberger’s software Geoframe. Two types of color designation are displayed in electrical image logs^54,55^. The color range is normalized over the entire intervals in the statically normalized image, whereas in the dynamically normalized image, the color range is normalized over a sliding window of 0.6096 m (2 ft). Core observations were calibrated with image logs to help fracture detection^55^.

Scanning electron microscope (SEM) analysis was conducted on the fresh surfaces of the core plug samples which are coated with gold to help detect the micropores associated with authigenic clay minerals. High-resolution fractures can also be identified by SEM imaging analysis^56^. Thin sections, which were impregnated with blue epiflourescent epoxy, were analyzed under plane-polarized and cross-polarized light to determine intergranular and intragranular pores at Houston Advanced Technology Center of Core Laboratory. Epifluorescence analysis was also performed on the same view of thin section observations.

Routine rock properties analysis (porosity and air permeability) was performed on the 30 full-diameter core sample under net confining pressure of 5.52 MPa, 13.66 MPa and 21.79 MPa respectively^36^. The CMS-300 instrument was used for the porosity measurements along with the permeability to gas measurements. Porosity was firstly determined for each sample by placing it into a stainless steel matrix cup, then pressure was vented at a known rate and unsteady-state Klinkenberg permeability was determined by pressure decay^36^. The core segment samples were then scanned by the Industrial X-ray Computed Tomography Facility at Houston Advanced Technology Center of Core Laboratory, which is described in detail at the website (http://www.corelab.com/ps/ct-scan-applications). A total of 30 cylindrical core samples were scanned by the ICT to analyze the fracture systems. Core Segments are collected from the following wells in the Kuqa depression of Tarim Basin in West China: KS 2-1-5; KS 2-2-3; KS 2-2-4; KS 2-2-5; and KS 208. The sample depth ranges from 6599.3 m to 6805.28 m. The cylindrical core segments have a radius of 50.9 mm. The lengths of these core segments have a wide range from 137.4 mm to 280.8 mm (Supplementary Table 1).

## Electronic supplementary material

Supplementary Table 1
