# Author Correction: Three-dimensional quantitative fracture analysis of tight gas sandstones using industrial computed tomography

**DOI:** 10.1038/s41598-018-28391-0

**Published:** 2018-07-11

**Authors:** Jin Lai, Guiwen Wang, Zhuoying Fan, Jing Chen, Ziqiang Qin, Chengwen Xiao, Shuchen Wang, Xuqiang Fan

**Affiliations:** 1 State Key Laboratory of Petroleum Resources and Prospecting, China University of Petroleum-Beijing, Beijing, 102249 China; 2 College of Geosciences, China University of Petroleum-Beijing, Beijing, 102249 China; 3 Research Institute of Petroleum Exploration and Development, Tarim Oilfield Company, CNPC, Korla, 841000 Xinjiang China; 4 Department of Petroleum Engineering, University of Wyoming, E Lewis Street, Engineering Building, Laramie, Wyoming, 82071-2000 USA

Correction to: *Scientific Reports* 10.1038/s41598-017-01996-7, published online 12 May 2017

In the original version of this Article, Affiliations 1 and 2 were not listed in the correct order. The correct affiliations are listed below:

Affiliation 1

State Key Laboratory of Petroleum Resources and Prospecting, China University of Petroleum-Beijing, 102249, China

Affiliation 2

College of Geosciences, China University of Petroleum-Beijing, 102249, China

This error has now been corrected in the PDF and HTML versions of the Article, and in the accompanying Supplementary Table file.

