# Supplementary material for: Three-dimensional quantitative fracture analysis of tight gas sandstones using industrial computed tomography
Source: Sci Rep. 2017 May 12;7:1825. doi: 10.1038/s41598-017-01996-7 (PMC5431860; doi:10.1038/s41598-017-01996-7)
Supplement: Supplementary file 1 — Supplementary Table 1 [file 41598_2017_1996_MOESM1_ESM.doc]

**Three-dimensional quantitative fracture analysis of tight gas sandstones using industrial computed tomography**

Jin Lai ^1,2*^ , Guiwen Wang^1,2^, Zhuoying Fan^1^, Jing Chen^1^, Ziqiang Qin^4^, Chengwen Xiao^3^, Shuchen Wang^1^, Xuqiang Fan^1^

Affiliations

1. State Key Laboratory of Petroleum Resources and Prospecting, China University of Petroleum-Beijing, 102249, China

2. College of Geosciences, China University of Petroleum-Beijing, 102249, China

3. Research Institute of Petroleum Exploration and Development, Tarim Oilfield Company, CNPC, Korla 841000, Xinjiang, China

Chengwen Xiao

4. Department of Petroleum Engineering, University of Wyoming, E Lewis Street, Engineering Building, Laramie, Wyoming 82071-2000, USA

Ziqiang Qin

Supplementary Table.1 Summarization of fracture parameters derived from a combination of 2-D slice analysis and 3-D visualization and counting.

| Sample | Depth (m) | Volume. of Interest | | 3D Volume rendering | | | | | | 2D Slice Measurements | | | | | | | High Density Measurement | | | | | |
| --- | --- | --- | --- | --- | --- | --- | --- | --- | --- | --- | --- | --- | --- | --- | --- | --- | --- | --- | --- | --- | --- | --- |
|  |  | Numbers of slices | Volume ROI (mm^3^) | Fracture Volume (mm^3^) | Total scanned area(mm^2^) | Numbers of Fracture | Average fracture volume (mm^3^) | Fracture porosity (%) | Fracture aperture (μm) | Numbers of Measurements | Average Length (mm) | Average aperture (mm) | Total Area Analzed (mm^2^) | Total Area of Fractures(mm^2^) | Fracture porosity (%) | Fracture Density (m^-1^) | HighDen Volume (mm^3^) | Total scanned area (mm^2^) | Numbers of Objects.n | Average Volume (mm^3^) | Fracture porosity (%) | Fracture aperture (μm) |
| 1 | 6713.61 | 739 | 287,802 | 3,735 | 9704 | 165 | 23 | 1.30 | 384.9 | 1501 | 2.8 | 1.7 | 959341 | 12478 | 1.30 | 4.38 | 1,908 | 15,627 | 209 | 9.1 | 0.66 | 122.1 |
| 2 | 6715 | 785 | 305,717 | N/A | N/A | N/A | N/A | 0.00 | 0.0 | N/A | N/A | N/A | 1019056 | N/A | 0.00 | 0.00 | 11,790 | 113,998 | 950 | 12 | 3.86 | 103.4 |
| 3 | 6722.3 | 751 | 292,476 | N/A | N/A | N/A | N/A | 0.00 | 0.0 | N/A | N/A | N/A | 974919 | N/A | 0.00 | 0.00 | 2,462 | 30,610 | 76 | 32.4 | 0.84 | 80.4 |
| 4 | 6722.66 | 590 | 229,774 | 2,705 | 5384 | 32 | 85 | 1.18 | 502.3 | 557 | 3.7 | 2.1 | 765915 | N/A | 1.18 | 2.66 | 5,258 | 89,988 | 101 | 52.1 | 2.29 | 58.4 |
| 5 | 6723.05 | 560 | 184,508 | 99 | 536.5 | 27 | 4 | 0.05 | 184.0 | 102 | 2.5 | 1.5 | 615025 | 330 | 0.05 | 0.42 | 11,827 | 273254 | 22 | 537.6 | 6.41 | 43.3 |
| 6 | 6734.83 | 749 | 291,697 | 664 | 1806 | 19 | 35 | 0.23 | 367.6 | 174 | 3.4 | 2.1 | 972323 | 2215 | 0.23 | 0.60 | N/A | N/A | N/A | N/A | 0.00 | 0.0 |
| 7 | 6738.39 | 803 | 312,727 | 1,659 | 3167 | 40 | 41 | 0.53 | 524.0 | 275 | 3.5 | 2.5 | 1042423 | 5535 | 0.53 | 0.92 | 825 | 6,184 | 103 | 8.0 | 0.26 | 133.4 |
| 8 | 6742.09 | 852 | 331,810 | N/A | N/A | N/A | N/A | 0.00 | 0.0 | N/A | N/A | N/A | 1106033 | N/A | 0.00 | 0.00 | 2,434 | 21,808 | 237 | 10.3 | 0.73 | 111.6 |
| 9 | 6743.21 | 697 | 271,445 | N/A | N/A | N/A | N/A | 0.00 | 0.0 | N/A | N/A | N/A | 904818 | N/A | 0.00 | 0.00 | 2,146 | 20,192 | 193 | 11.1 | 0.79 | 106.3 |
| 10 | 6805.28 | 588 | 228,996 | 609 | 3166 | 101 | 6 | 0.27 | 192.3 | 1052 | 2.3 | 1.1 | 763319 | 2037 | 0.27 | 3.23 | 868 | 8,421 | 69 | 12.6 | 0.38 | 103.1 |
| 11 | 6767.51 | 502 | 195,503 | N/A | N/A | N/A | N/A | 0.00 | 0.0 | N/A | N/A | N/A | 651677 | N/A | 0.00 | 0.00 | 5,993 | 5,298 | 47 | 127.5 | 3.07 | 1131.2 |
| 12 | 6768.49 | N/A | N/A | N/A | N/A | N/A | N/A | 0.00 | 0.0 | N/A | N/A | N/A | N/A | N/A | 0 | 0 | N/A | N/A | N/A | N/A | 0.00 | 0.0 |
| 13 | 6769.97 | 523 | 203,681 | 1,014 | 4296 | 23 | 44 | 0.50 | 236.1 | 597 | 6.6 | 1.1 | 678938 | 3390 | 0.50 | 5.82 | N/A | N/A | N/A | N/A | 0.00 | 0.0 |
| 14 | 6770.55 | N/A | N/A | N/A | N/A | N/A | N/A | 0.00 | 0.0 | N/A | N/A | N/A | N/A | N/A | 0 | 0.00 | N/A | N/A | N/A | N/A | 0.00 | 0.0 |
| 15 | 6769.44 | 458 | 178,367 | 36 | 223.4 | 9 | 4 | 0.02 | 160.6 | 39 | 3.4 | 0.9 | 594558 | 121 | 0.02 | 0.22 | N/A | N/A | N/A | N/A | 0.00 | 0.0 |
| 16 | 6775.81 | 463 | 180,315 | 98 | 638.4 | 36 | 3 | 0.05 | 153.4 | 231 | 2.2 | 0.8 | 601048.5 | 332.010 | 0.06 | 0.84 | 1,164 | 21,417 | 20 | 58.2 | 0.65 | 54.3 |
| 17 | 6674.64 | 936 | 364,524 | 6,781 | 19375 | 413 | 16 | 1.86 | 350.0 | 3592 | 2.5 | 1.6 | 1215079 | 22678 | 1.87 | 7.52 | 777 | 7,104 | 71 | 10.9 | 0.21 | 109.4 |
| 18 | 6680.42 | 707 | 275,340 | 307 | 1667 | 94 | 3 | 0.11 | 184.3 | 433 | 2.4 | 1.2 | 917800 | 1033 | 0.11 | 1.14 | 25,175 | 387,930 | 715 | 35 | 9.14 | 64.9 |
| 19 | 6682.38 | 755 | 294,033 | 12,728 | 29803 | 195 | 65 | 4.33 | 427.1 | 2801 | 9.1 | 2.0 | 980111 | 42474 | 4.33 | 25.93 | 596 | 3,695 | 112 | 5.3 | 0.20 | 161.2 |
| 20 | 6684.35 | 717 | 279,234 | N/A | N/A | N/A | N/A | 0.00 | 0.0 | N/A | N/A | N/A | 930781 | N/A | 0.00 | 0.00 | 672 | 6,309 | 62 | 10.8 | 0.24 | 106.6 |
| 21 | 6700.36 | 703 | 273,782 | N/A | N/A | N/A | N/A | 0.00 | 0.0 | N/A | N/A | N/A | 912607 | N/A | 0.00 | 0.00 | 425 | 3,203 | 57 | 7.4 | 0.16 | 132.6 |
| 22 | 6709.3 | 825 | 321,295 | 133 | 689.7 | 29 | 5 | 0.04 | 192.6 | 218 | 1.9 | 1.1 | 1070983 | 449 | 0.04 | 0.38 | 6,746 | 87,714 | 323 | 20.9 | 2.10 | 76.9 |
| 23 | 6718.42 | 900 | 350,503 | 1,992 | 8014 | 154 | 13 | 0.57 | 248.5 | 2323 | 2.9 | 1.2 | 1168345 | 6659 | 0.57 | 5.86 | 9,963 | 119,050 | 351 | 28 | 2.84 | 83.7 |
| 24 | 6723.2 | 922 | 359,071 | 2,614 | 7623 | 91 | 29 | 0.73 | 342.9 | 1277 | 3.9 | 1.2 | 1196904 | 8731 | 0.73 | 4.16 | 8,251 | 136,876 | 136 | 60.7 | 2.30 | 60.3 |
| 25 | 6725.71 | 804 | 313,116 | 1,999 | 5764 | 27 | 74 | 0.64 | 346.7 | 675 | 9.6 | 1.2 | 1043721 | 6671 | 0.64 | 6.21 | 2,820 | 33,480 | 69 | 40.9 | 0.90 | 84.2 |
| 26 | 6599.3 | 680 | 264,825 | N/A | N/A | N/A | N/A | 0.00 | 0.0 | N/A | N/A | N/A | 882749 | N/A | 0.00 | 0.00 | 39,156 | 913,661 | 364 | 107.6 | 14.79 | 42.9 |
| 27 | 6601.35 | 836 | 325,579 | N/A | N/A | N/A | N/A | 0.00 | 0.0 | N/A | N/A | N/A | 1085263 | N/A | 0.00 | 0.00 | 1,556 | 11,156 | 221 | 7 | 0.48 | 139.4 |
| 28 | 6603.28 | 833 | 324,410 | N/A | N/A | N/A | N/A | 0.00 | 0.0 | N/A | N/A | N/A | 1081368 | N/A | 0.00 | 0.00 | 3,063 | 31,453 | 431 | 7.1 | 0.94 | 97.4 |
| 29 | 6606.07 | N/A | N/A | N/A | N/A | N/A | N/A | 0.00 | 0.0 | N/A | N/A | N/A | N/A | N/A | 0.00 | 0.00 | N/A | N/A | N/A | N/A | 0.00 | 0.0 |
| 30 | 6609.52 | 491 | 191,219 | N/A | N/A | N/A | N/A | 0.00 | 0.0 | N/A | N/A | N/A | 637397 | N/A | 0.00 | 0.00 | 506 | 10,179 | 5 | 101.2 | 0.26 | 49.7 |
